# Effects of permethrin on ambrosia beetles (Coleoptera: Curculionidae: Scolytinae) in ornamental nurseries

**DOI:** 10.1093/jisesa/iead052

**Published:** 2023-07-07

**Authors:** Zia V Williamson, Brett R Blaauw, Shimat V Joseph

**Affiliations:** Department of Entomology, The University of Georgia, 1109 Experiment Street, Griffin, GA 30223, USA; Department of Entomology, The University of Georgia, 413 Biological Science, 120 Cedar Street, Athens, GA 30602, USA; Department of Entomology, The University of Georgia, 1109 Experiment Street, Griffin, GA 30223, USA

**Keywords:** Xylosandrus, Xylosandrus crassiusculus, verbenone, field nursery

## Abstract

Exotic ambrosia beetles (Coleoptera: Curculionidae: Scolytinae), such as *Xylosandrus crassiusculus* (Motschulsky), *Xylosandrus germanus* (Blandford), and *Xylosandrus compactus* (Eichoff) are serious pests in southeastern ornamental nurseries. Preventative pyrethroid trunk sprays effectively reduce boring damage. However, it is unclear how pyrethroids such as permethrin prevent attack. Thus, the objective was to determine how permethrin-treated bolts interact with invading ambrosia beetles. In 2022, a study with 2 independent trials was conducted in a nursery on red maple (*Acer rubrum* L.), bolts during March and April, respectively. The treatments were (i) nonbaited, nontreated bolt, (ii) ethanol baited bolt, (iii) nonbaited bolt + glue [painted on bolt], (iv) ethanol baited bolt + glue, (v) ethanol baited bolt + glue + permethrin, (vi) ethanol baited bolt + glue + permethrin + verbenone, and (vii) ethanol baited bolt + glue + verbenone. Ambrosia beetles trapped on glue, beetles which fell into the pail with soap solution under the bolts, and entry holes on bolts were quantified. Permethrin prevented beetle attacks but did not reduce the number of ambrosia beetles landing on the treated bolts. Verbenone reduced ambrosia beetles from landing on the bolts but did not prevent boring into bolts. The numbers of ambrosia beetles in soapy water were not significantly different among treatments. Ambrosia beetles are landing on permethrin-treated bolts but not boring into the bolts, implying that fresh permethrin residues may not be necessary for ambrosia beetle management.

Invasive ambrosia beetles (Coleoptera: Curculionidae: Scolytinae) are serious pests of ornamental tree nurseries in the southeastern United States (Rabaglia et al. 2006, Fulcher et al. 2012). Among them, *Xylosandrus* species, such as the granulate ambrosia beetle, *Xylosandrus crassiusculus* (Motschulsky), the black stem borer, *Xylosandrus germanus* (Blandford), and the black twig borer, *Xylosandrus compactus* (Eichoff) are the most damaging pests (Ranger et al. 2016b, Gugliuzzo et al. 2021, Monterrosa et al. 2022). Georgia ornamental nursery production was valued at $444 million USD in 2020 (UGA 2020). Millions of dollars in losses occur in the Georgia nursery industry as a result of nonsalable plants affected by arthropod pests and pest management expenditures (LeBude et al. 2012). Because of this, it is important to understand the modes of exposure and action of specific management tactics, such as insecticides.

*Xylosandrus* species typically have 3 generations per year in the southeastern United States; however, this is affected by abiotic factors such as rain and air temperature (Frank et al. 2013, Ranger et al. 2016b). *Xylosandrus* spp. larvae develop from eggs laid by foundress females, which bore and inoculate galleries with symbiotic fungi stored in their mycangium (Weber and McPherson 1983, Ranger et al. 2016a). Females use stress signals, especially ethanol, to locate and attack stressed trees (Ranger et al. 2010, 2015). Adult females mate and overwinter inside the galleries (Weber and McPherson 1983, Gugliuzzo et al. 2021). Mated females emerge from those galleries beginning in early spring (Weber and McPherson 1983, Greco and Wright 2015, Ranger et al. 2016a).

Because adults and larvae of *Xylosandrus* spp. feed only on the symbiotic fungi and do not consume tree tissues, insecticides with a systemic mode of exposure are ineffective, and preventative contact exposure of insecticides is essential for management (Ranger et al. 2016b). Pyrethroids are often used as preventative trunk sprays in early spring before and during peak flights (Frank and Sadof 2011, Reding et al. 2013, Ranger et al. 2016a). Repeated applications of pyrethroids, particularly permethrin or bifenthrin, are recommended between 8 and 17 d intervals (Brown et al. 2020), although a consistent efficacy in preventing ambrosia beetle infestation is still not guaranteed (Ranger et al. 2016a). Other insecticide chemistries have been tested as preventative trunk sprays, but none have provided satisfactory efficacy against *Xylosandrus* spp. to date (Joseph et al. 2022a, 2022b). Currently, preventative applications of pyrethroids, especially bifenthrin and permethrin, are the only insecticide option for ambrosia beetle management. Despite this, other noninsecticide management approaches were explored (Rivera et al. 2020, Agnello et al. 2021).

Verbenone is a known repellent as it repels bark beetles, serving as an anti-aggregation pheromone for species such as *Dendroctonus ponderosae* Hopkins*, Dendroctonus frontalis* Zimmerman, and *Ips typographus* L. (Pitman and Vité 1969, Pitman et al. 1969, Lindgren and Miller 2002). This compound is produced by fungal symbionts of various bark beetle species, such as *Ips typographus* L. and *D. frontalis*, which oxidize trans-verbenol to verbenone (Brand et al. 1976, Leufvén et al. 1984). Previously, verbenone has been evaluated on ambrosia beetles, including *Xylosandrus* spp. with variable results (Lindgren and Miller 2002, Rivera et al. 2020, Agnello et al. 2021). Verbenone reduced sticky trap captures of the redbay ambrosia beetle, *Xyleborus glabratus* Eichoff (Hughes et al. 2017). Similarly, the verbenone dispenser reduced attacks on trap trees, but attacks occurred on all trees (Ranger et al. 2021). Verbenone was included in the current study as a known repellent to compare the behavior of approaching ambrosia beetles to permethrin-treated bolts.

Although pyrethroids are widely used for preventing ambrosia beetle attacks, it is unclear how these pyrethroid residues effectively reduce the *Xylosandrus* spp. from boring into the bark and whether pyrethroids prevent the landing of adult beetles. While studies evaluating modes of repellent behavior of ambrosia beetles to permethrin are limited, the mode of repellency has been explored for many other arthropod pests, with variable results in terms of whether contact or noncontact repellency is the proposed mode of action (Dobrin and Hammond 1985, Lowery and Boiteau 1988, Lin et al. 1993, Eisen et al. 2017). Therefore, there is a knowledge gap in understanding the repellency behavior of ambrosia beetles approaching pyrethroid-treated tree trunks. We hypothesize that pyrethroids, especially permethrin, prevent ambrosia beetle attacks through repellency, although it is unclear if noncontact (not landing on the permethrin-treated surface) or contact (leaving the surface after landing on the permethrin-treated surface) or other mechanisms, such as mortality are involved. The objective was to determine the mechanism that causes reduced attacks on permethrin treated trees. This knowledge will help researchers and growers improve the management of *Xylosandrus* spp. by increasing their understanding of permethrin’s effect on ambrosia beetles and corresponding methods of ambrosia beetle management. Thus, this study seeks to expand knowledge regarding the successful implementation of preventative pyrethroid sprays in ornamental nurseries, as well as aid in offering better recommendations for growers.

## Materials and Methods

### Study Site

In 2022, a study was conducted at an in-ground tree nursery in Lamar County, Georgia, USA. The site consists of ~35 ha in production, and trees were spaced ~2 m apart. The experiment was employed on the western edge of the nursery. The traps were deployed along the edge of the woodline. The experimental area was surrounded by the trees grown in the nursery and mixed hardwood and pine forest. Common trees grown in the nursery consisted largely of deciduous ornamental trees. Forest trees were at least 5 m away from the edge of the woodline. The site was managed following standard production recommendations and commercial pesticide management guidelines. Pesticides were not used for the duration of the experiment near the experimental area.

### General Approach

Wooden bolts were utilized to determine the landing and boring behavior of ambrosia beetles in the field nurseries. This technique has been used in previous studies and is an efficient methodology for evaluating ambrosia beetle damage (Klingeman et al. 2017, Reding and Ranger 2020, Cavaletto et al. 2021). The bolts were cored in the center and 90% ethanol was added to the cores to attract beetles to the bolts. This concentration was selected because higher ethanol concentrations were linked to higher attacks from scolytines (Cavaletto et al. 2021, Caveletto et al. 2023). Half of the surface area of the bolts was coated with glue to trap those ambrosia beetles if they landed on the bolts. Plastic pails with soap water were placed underneath the bolts to trap those beetles that fell after contacting the bolts. Some bolts were sprayed with permethrin and others received repellent verbenone dispensers to document the behavior of approaching ambrosia beetles to permethrin. The numbers of entry holes and ambrosia beetles trapped in the glue or fallen into the pail were quantified. The relative number of beetles stuck on the glue with and without permethrin suggests whether permethrin repelled ambrosia beetles approaching the treated bolts. Those beetles collected in the pail indicate whether the beetles were intoxicated and dropped after contacting the permethrin residues applied on the bolts. The relative numbers of entry holes suggest whether the normal behavior of beetles changed after contacting the permethrin-treated and nontreated bolts.

### Bolt Trap

**Fig. 1.**
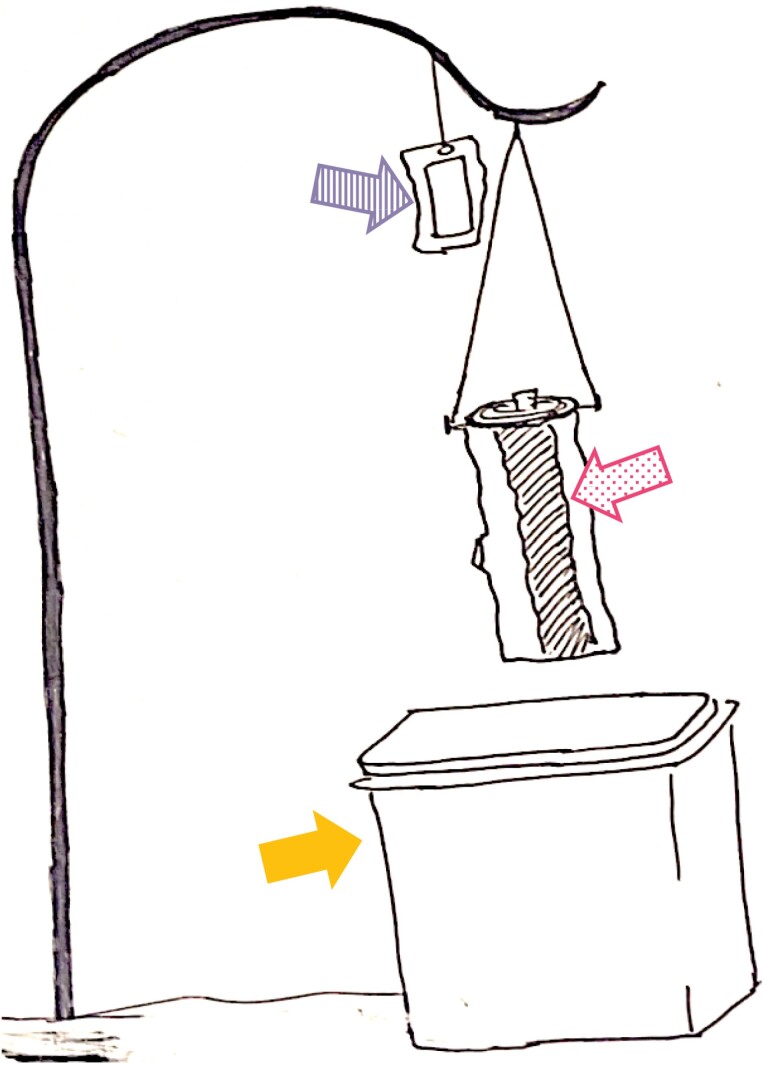
Set up of wooden bolt with glue bands (dotted arrow) painted on the bolt and verbenone pouch (striped arrow) suspended from the shepherd’s hook in a field nursery. Permethrin was trunk sprayed before the glue was painted on the bolt. A 18.9 L pail (solid arrow) with soap solution was placed below the bolt to collect the dropping ambrosia beetles.

Thirty-five bolts were prepared using *Acer rubrum* L. branches from residential yards in Fayette and Henry Counties in Georgia. No insecticide applications were made to these trees prior to obtaining them for the study. This tree species was selected for the experiment because *Xylosandrus* spp. routinely attack this tree species, and it is an important tree species in many nurseries in the southeastern United States (Frank et al. 2013, USDA 2017). The branches were cut into 50 cm long pieces and temporarily stored in the refrigerator for ~7 d before deployment. These bolts were 5.1–7.3 cm in diameter. Before deployment for the experiment, the bolts were further cut into 25 cm long pieces. A 1.5 × 7 cm (diameter × deep) hole was drilled on the top of each bolt using a handheld drill. All bolts, even those treated without ethanol, had the drilled hole sealed with a 1.25 cm (diameter) natural cork stopper. Two screws were affixed to the top of the bolts, and a 50 cm long string was then tied to the screws (Fig. 1). The bolts were suspended from a 122 cm metal shepherd’s hook. Bolts were hung at ~91 cm above the ground. Previous studies showed that captures and attacks of ambrosia beetles were greatest closer to the ground, with more than 90% of attacks from *X. germanus* when positioned within 1 m from the ground (Reding et al. 2010, Klingeman et al. 2017). One bolt was suspended from each shepherd’s hook.

### Experimental Design and Treatments

Experiments were conducted in the spring when the *Xylosandrus* spp. adults were actively flying (Monterrosa et al. 2022). The first repetition of the experiment was conducted from 2 March to 16 March (trial 1) and the experiment was repeated from 2022 11 April to 25 April (trial 2) in the same nursery. Both trials of the experiment operated independently, with bolt traps being removed and replaced with new ones between trials. Treatments are listed as follows, with abbreviations in parenthesis: (i) nonbaited, nontreated bolt [none], (ii) ethanol baited bolt [E], (iii) nonbaited bolt + glue [G], (iv) baited bolt + glue [E+G], (v) baited bolt + glue + permethrin [E+G+P], (vi) baited bolt + glue + permethrin + verbenone [E+G+P+V], and (vii) baited bolt + glue + verbenone [E+G+V]. The treated bolts were arranged in a randomized complete block design with 5 replications. The bolts were deployed 10 m apart along the woodline about 0.5 m inside the nursery from the edge. Blocks of 7 bolts were spaced 20 m apart.

The none and E bolts were included to ensure the attraction of *Xylosandrus* spp. to the ethanol bait. To ensure glue by itself was not attracting the adult ambrosia beetles, a G treatment was added with only glue painted on bolts with and without ethanol bait. To explore the repellent effects of permethrin and verbenone, the E+G, E+G+P and E+G+V treatments were compared.

For treatments with ethanol, 10 ml of 90% ethanol was poured into the cored hole at the top of the bolt at set up and at the 6 (trial 1) or 7 d (trial 2) of the experiment. The cork was not wrapped with any material to prevent ethanol volatilization. For bolts that received glue as a part of their designated treatment, glue was painted lengthwise, in 2.5 cm wide strips, directly onto the designated bolts using a stiff bristled paintbrush at the start of the trial. The glue used in the study to capture ambrosia beetles was deemed weather- and rain-resistant by the manufacturer (Pestick, Phytotronics, Inc., Earth City, Missouri, USA). The coverage area was 1:1 for glue: nonglue on the bolt surface so that there is a sufficient nonglued surface for ambrosia beetles to land. Thus, there was also 2.5 cm wide of exposed bark (no glue) areas between each glue section (Fig. 1). The number of glue strips on the bolts varied from 3 to 5 strips as the circumference of the maple bolts varied; however, the percentage of area covered remained the same regardless of bolt size. For permethrin-treated bolts, the glue was applied after applying permethrin for certain treatments, as indicated in the treatment descriptions.

As described previously, an insecticide solution was sprayed on certain treatment groups to determine the repellent effects of pyrethroids. The insecticide used in the experiment was permethrin (Perm-UP 3.2 EC, 36.8% permethrin; FMC Corporation, Philadelphia, Pennsylvania, USA). Permethrin was applied at the start of the trial before the glue was applied to certain treatments. The application rate of Perm-UP 3.2 EC was 584 ml per ha. The prepared insecticide solution was sprayed on the bolts after suspending them by their hangers from a PVC pipe for uniform coverage of insecticide until run-off. Insecticide solution was applied using a CO_2_-powered single boom handheld sprayer at 206.8 kPa. The nozzle was attached with a TeeJet 8002VS (yellow-colored tip, TeeJet Technologies, Glendale Heights, Illinois, USA). For appropriate treatments, a single verbenone pouch was placed on the shepherd’s hook that the bolt hung on at the start of the trial, 5 cm above the bolts (Verbenone, Synergy Shield Verbenone pouches, 97.0% verbenone; Synergy Semiochemical Corporation, Delta, British Columbia, Canada) (Fig. 1). The verbenone pouch remained in place for the entirety of the trial, and was not replaced.

Because ambrosia beetle adults that land on permethrin-treated bolts may become moribund or die, adults could fall. If the killing was happening due to contact with permethrin, we would expect to see more beetles in the pail under permethrin-treated bolts. To capture the ambrosia beetle adults falling from the bolts, a 15.1-liter plastic pail containing 60 ml of soap (Proctor and Gamble, Kansas City, Kansas, USA) and 1 liter of water that was prepared as a solution was placed under each bolt (all treatments included) (Fig. 1). The soap solution was emptied and refilled at every observation date.

### Evaluation

Bolts were evaluated at 2, 6, and 14 d for trial 1, and 2, 7, and 14 d in trial 2. There was a heavy rain forecast on the fifth day of trial 2 (Supplementary Fig. 1), which may have affected beetle flight and their interactions with the bolts. The cumulative number of ambrosia beetle entry holes on the bolts was quantified at each evaluation date. The entry holes were circled using a different colored wax pencil at each check date to avoid double counting. Beetles that bored into the bolts were not excised. All ambrosia beetles stuck on the glue were counted at each evaluation day post-treatment. At the end of the trial, all bolts were collected and placed in plastic bags, transported to the laboratory, and stored in a freezer at −18°C until processing. All ambrosia beetles, including non-*Xylosandrus* spp., were counted, as identifying them was challenging in the field. The cumulative data up to the 14 d check date are presented by adding the number of beetles in the glue, the number of beetles in the pail (in soap), and the number of entry holes up to 14 d post-treatment.

Before processing, bolts were allowed to thaw at 21°C for 2 h. The ambrosia beetles caught on the glue were removed from the bolts by painting Histo-Clear (Electron Microscopy Sciences, Hatfield, Pennsylvania, USA) onto the glue with a paintbrush. After 2 min, ambrosia beetles loosen enough to be removed using pointed forceps. The removed adults were placed into a vial filled with 10 ml of Histo-Clear. After 15 h in the solvent, adults were rinsed in water and 70% ethanol before storage in a vial with 70% ethanol for identification at a later date. Those ambrosia beetles inside the galleries within the bolts were not extracted.

Adult ambrosia beetles collected in soapy water under the bolts were screened by pouring it through a mesh bag and recovering the contents at the 3 evaluation dates. From filtered samples, the ambrosia beetles were sorted and removed using a paintbrush and stored in microcentrifuge tubes filled with 70% ethanol for identification. In a few cases, soap samples of individual bolts were lost due to rain, and sustained wind. Those samples were considered missing data points. Ambrosia beetles were stored in ethanol and then identified at a later date. Beetles were identified to species using Bateman and Hulcr’s guide to bark and ambrosia beetles (Bateman and Hulcr 2017). *Xylosandrus* were identified to species, with all other ambrosia beetle genera being grouped into the “other” category. Thus, *Xylosandrus* was presented as the primary genus of concern for southeastern ornamental nurseries (Ranger et al. 2016b, Gugliuzzo et al. 2021, Monterrosa et al. 2022).

### Statistical Analyses

All statistical analyses were conducted utilizing R software (R Core Team 2022). Count data for cumulative numbers of entry holes, cumulative numbers of beetles in soap, and the cumulative number of beetles captured in glue at 14 d post-treatment, were square-root transformed. One-way analysis of variance (ANOVA) of data using the aov() function using treatment and block as parameters were conducted. Tukey’s honest significant difference (HSD) test was then conducted using the Tukey HSD() function of *R* for a post hoc comparison of means.

Because the study’s primary focus is to determine the mechanism of how ambrosia beetles respond to permethrin residues on bolt surfaces, ANOVA was conducted, excluding the nonglue treatments, as they may disproportionately bias the outcome of adult beetle entry holes. Thus, the no ethanol and ethanol only (no glue treatments) treatments were excluded from the analysis. Data were analyzed separately for each of the 2 trials.

## Results

**Fig. 2.**
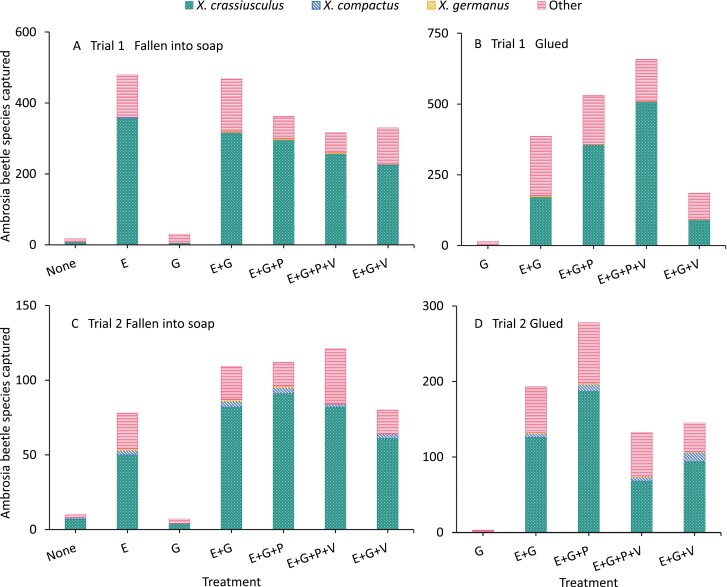
Cumulative captures of ambrosia beetles by species in (A, C) soap solution in pails and (B, D) glue painted on bolts for trial 1 (A, B) and trial 2 (C, D). Abbreviations: E, Ethanol; G, Glue; P, Permethrin; and V, Verbenone. The other ambrosia beetles collected were *Ambrosiudmus* spp., *Cyclorhipidion* spp., *Hypothenemus* spp., *Xyleborinus* spp., and *Xyleborus* spp. were labeled “other”.

**Fig. 3.**
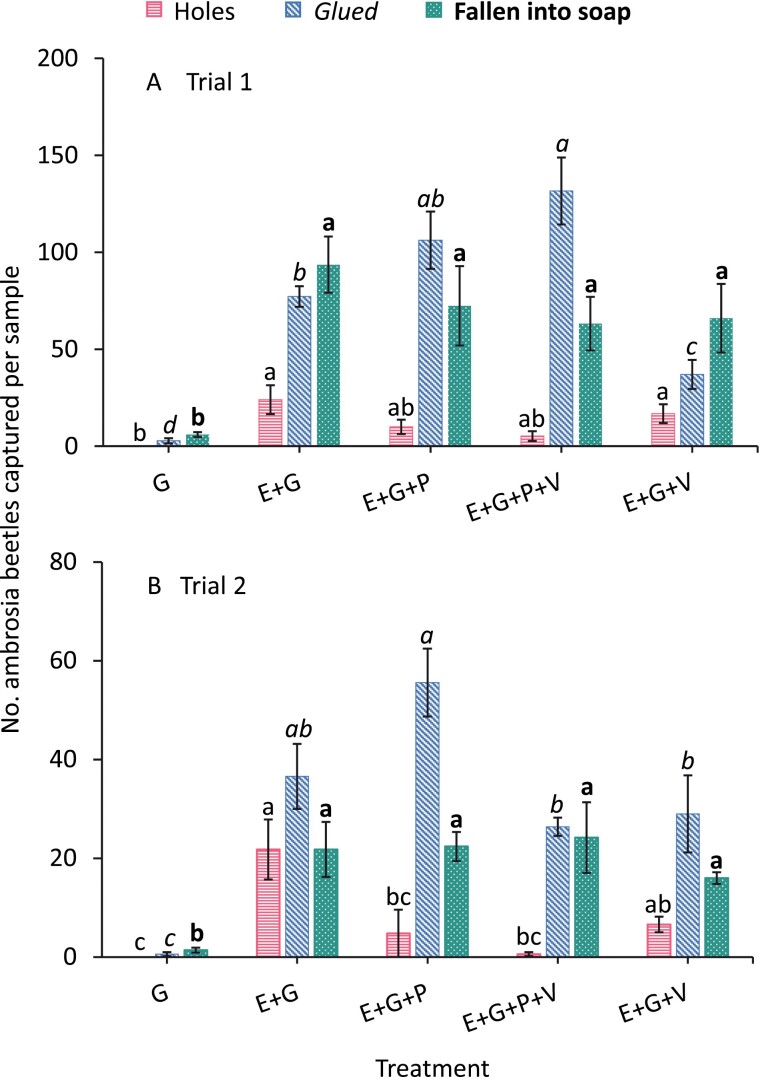
Mean ± (SE) number of ambrosia beetles collected on various treatments for (A) trial 1, and (B) 2 at 14 d post-treatment. Bars with the same letter types (regular, italics, and bold fonts) were compared among treatments and the same letters among treatments are not significantly different (Tukey’s HSD Test, α = 0.05). Abbreviations: E, Ethanol; G, Glue; P, Permethrin; and V, Verbenone.

Most beetles captured in the glue on the bolts and soapy water under the bolts were *Xylosandrus* spp. (74% of 2002 and 77% of 517 beetles captured in trials 1 and 2, respectively) (Fig. 2). Specifically, most of the *Xylosandrus* spp. beetles captured were *X. crassiusculus* (73% of 2002 and 73% of 517 beetles captured in trials 1 and 2, respectively). Other genera captured include *Ambrosiudmus*, *Cyclorhipidion*, *Hypothenemus*, *Xyleborinus*, and *Xyleborus* spp. In addition, low numbers of *X. compactus* and *X. germanus* were captured. During the trials, weather conditions were variable, such as intermittent frost and heavy rain (Supplementary Fig. 1). Ambrosia beetles that were not attracted to bolts only coated with glue and were attracted to ethanol-infused bolts in both trials (Fig. 3).

### Entry Holes

For trial 1, the numbers of holes between the E+G and E+G+P treatments were not significantly different (*F* = 6.3, df = 4, *P* = 0.003; Fig. 3A). The number of holes for the E+G+V treatment was not significantly different from the E+G treatment (Fig. 3A). For trial 2, the numbers of holes were significantly lower for the E+G+P and E+G+P+V treatments than for the E+G treatment, whereas there was no significant difference in numbers of holes between the E+G and E+G+V treatments (*F* = 10.1, df = 4, *P* < 0.001; Fig. 3B).

### Ambrosia Beetles in Glue and Soap

For trial 1, the numbers of beetles captured in glue were significantly greater for the E+G+P+V treatment than for the E+G and E+G+V treatments (*F* = 60.9, df = 4, *P* < 0.001; Fig. 3A). However, there was no significant difference in the number of beetles captured between the E+G+P+V and E+G+P treatments (Fig. 3A). In addition, the number of beetles in glue for the E+G+V treatment was significantly lower than for the E+G treatment (Fig. 3A). For trial 2, although the numbers of beetles captured in glue for the E+G+P treatment were not significantly different from the E+G treatment, the E+G+P treatment captured more numbers of beetles than the E+G+P+V and E+G+V treatments (*F* = 30.8, df = 4, *P* < 0.001; Fig. 3B). In both trials, the number of beetles collected in soap were significantly lower for the G treatment than for any other treatments (trial 1, *F* = 10.18, df = 4, *P* < 0.001; Fig. 3A; trial 2, *F* = 13.7, df = 4, *P* < 0.001; Fig. 3B). There were no significant differences between treatments excluding the G treatment in both trials.

## Discussion

*Xylosandrus crassiusculus* was the most commonly captured species of ambrosia beetle in the current study. It is unknown why this is the most common beetle captured, but this finding is consistent with previous southeastern studies where more than half of all ambrosia and bark beetles captured were *X. crassiusculus* (Ranger et al. 2016a, Monterrosa et al. 2022).Results show that the numbers of ambrosia beetles captured on glue-painted bolts treated with and without permethrin were not different, or in some cases, greater on bolts treated with permethrin than without permethrin. This suggests that ambrosia beetles landed on the permethrin-treated bolts and were not repelled by volatiles of permethrin applied in the bolts through noncontact repellency. In addition, the number of entry holes was lower on the permethrin-treated bolts than on the nontreated bolts in the second trial, but this difference was not observed in the first trial. Notably, the ambrosia beetle counts were much higher in the first trial than in the second. The data indicate that the underlying mechanism could be either contact repellency or intoxication after ambrosia beetle adults come in contact with permethrin-treated bolts. In a contact repellency scenario, those ambrosia beetle adults who land on the permethrin-treated bolts might have flown off after sensing permethrin residues instead of boring entrance holes. Previous studies show that when contact repellency was the leading mechanism, the damage was prevented by pests, such as *E. varivestis*, *M. persicae*, and *Stephanitis pyrioides* Scott (Hemiptera: Tingidae), as these organisms came in contact with permethrin-treated surfaces and then subsequently attempted to leave the permethrin-treated area or reduce feeding damage while displaying permethrin induced irritability (Dobrin and Hammond 1985, Lowery and Boiteau 1988, Joseph 2020). The other possibility is that they were intoxicated and became moribund or dead after contact with permethrin residues and knocked down to the ground. Because the ambrosia beetles collected in the pail with soap solution were similar numbers with and without permethrin application, we cannot determine whether beetles fell into the pail due to permethrin exposure. Based on the data, it is clear that the soapy water pail did not attract adult ambrosia beetles. Because data show that beetles landed on permethrin-treated bolts and did not bore extensively, but there was no difference in the beetles collected in the pail, more controlled studies are warranted to determine the fate of ambrosia beetle adults who land on permethrin-treated surfaces. It is possible that some beetles attempting to land on the bolt bounced off after hitting the bolts and fell into the pail.

Although fewer holes were found on permethrin-treated bolts, more beetles were captured in the glue compared to bolts without permethrin application. The exact reason is unclear. Pyrethroids are known to cause excitatory behavior when insects come in contact with residues. Pyrethroids act as sodium channel modulators, affecting the insect nervous system (IRAC 2022). Because the action potentials along nerves keep the sodium channels open, insects exhibit hypersensitivity to stimuli when exposed, causing excitation and irritation behaviors (Meyer and Shafer 2006, Yan et al. 2011). For example, there was clear evidence of excitatory reaction on permethrin-coated or impregnated surfaces in honey bees, diamondback moths, ticks, and mosquitoes, respectively (Rieth and Levin 1988, Lin et al. 1993, Faulde et al. 2008, Orsborne et al. 2016, Bowman et al. 2018). Other studies confirm this phenomenon, with mosquitoes and lace bugs exhibiting behavioral avoidance of pyrethroids, including permethrin, after contacting treated surfaces (Boonyuan et al. 2016, Joseph 2020). Because of this, it is possible that ambrosia beetles landed on the surface of the bolt and, upon exposure to permethrin, may have elicited excitatory, erratic movement while trying to flee the treated site, causing higher numbers of ambrosia beetles to be caught on the glue coated on permethrin-treated bolts.

In the current study, the verbenone dispenser reduced the number of ambrosia beetles on the glue but did not effectively reduce the entry holes on the bolts. Verbenone functions by interrupting the attraction of beetles to conspecific pheromones and host volatiles, such as ethanol (Ranger et al. 2013, 2021). A previous study showed that *X. germanus* trap captures were lower in verbenone-treated funnel traps, although verbenone did not reduce attacks on the tree, similar to what was seen in the current study (Dodds and Miller 2010). Furthermore, the range of action for verbenone is largely unknown, so it is possible that the effects of verbenone-treated bolts may have interfered with the bolts adjacent to them. Ranger et al. (2013) adopted 10 m between verbenone dispensers and attacks increased as the distance was more than 10 m. In the current study, a 10 m spacing between bolts was maintained and thus, interactions from adjacent verbenone dispensers are more likely to be minimal, but more future research is warranted to determine the active range of verbenone dispensers. In ornamental production, the tolerance for ambrosia beetle attacks is minimal, and inconsistent efficacy when only using verbenone for managing ambrosia beetles, as noted in current and previous studies, needs more future research. However, verbenone may serve as a component of integrated pest management programs when combined with other intervention methods, such as permethrin trunk sprays (Frank and Sadof 2011, Joseph 2022a, 2022b), warranting more research.

Understanding the mechanism of repellency of permethrin to ambrosia beetles has obvious implications for managing ambrosia beetles in nursery production. The results of the current study suggest that permethrin sprays deterred boring to some extent through contact repellency. Many nursery growers think that fresh residues of pyrethroid and their resultant volatiles prevent attacks from ambrosia beetles. The results of our study are in contention with that belief and show that multiple applications of pyrethroids at close intervals may be unnecessary. Brown et al. (2020) suggested that permethrin applied at 8–17 d intervals reduced adult beetle attacks. Thus, concurrent applications of permethrin at closer than 14 d offer no benefit, with increased volatiles not being as important as actual contact with permethrin residues. With concerns for harmful effects on natural enemies and pollinators resulting from permethrin application (Frank and Sadof 2011, LeBude et al. 2012), this information is important for ornamental growers to reduce pyrethroid exposure to nontargets.

In summary, permethrin did not stop ambrosia beetles from landing on treated bolts, but there is evidence that it may prevent successful boring attempts. Captures of ambrosia beetles on glue were equal or greater on permethrin-treated than nontreated bolts. Future research exploring the rate-dependent effects of permethrin deterrence to ambrosia beetles is warranted, with the potential for future lab-based experiments regarding the repellency of ambrosia beetles to permethrin to be conducted. Verbenone reduced the landing of ambrosia beetles on the ethanol-baited bolts but did not consistently deter ambrosia beetle from boring into the bolts. This suggests that a more thorough understanding of the interaction between ethanol emittance and verbenone on deterrence of approaching ambrosia beetles may provide valuable information regarding verbenone as a noninsecticide strategy. The results from the current study improved our understanding of how permethrin interacts with ambrosia beetles and prevents subsequent attacks.

## Supplementary Material

iead052_suppl_Supplementary_Figure_S1

## References

[CIT0001] Agnello AM , Combs DB, Filguerias CC, Willett DS, Mafra-Neto A. Reduced infestation by *Xylosandrus germanus* (Coleoptera: Curculionidae: Scolytinae) in apple trees treated with host plant defense compounds. J Econ Entomol. 2021:114:2162–2171. 34378779 10.1093/jee/toab153

[CIT0002] Bateman C , Hulcr J. A guide to Florida’s common bark and ambrosia beetles. UF/IFAS University of Florida; 2017. FOR 321:1–32. [accessed 22 Sep 2022]. https://edis.ifas.ufl.edu/pdf%5CFR%5CFR38900.pdf.

[CIT0003] Boonyuan W , Bangs MJ, Grieco JP, Tiawsirisup S, Prabaripai A, Chareonviriyaphap T. Excito-repellent responses between *Culex quinquefasciatus* permethrin susceptible and resistant mosquitoes. J Insect Behav. 2016:29(4):415–431. 10.1007/s10905-016-9570-4

[CIT0004] Bowman NM , Akialis K, Cave G, Barrera R, Apperson CS, Meshnick SR. Pyrethroid insecticides maintain repellent effect on knock-down resistant populations of *Aedes aegypti* mosquitoes. PLoS One. 2018:13(5):e0196410. 10.1371/journal.pone.0196410 29763445 PMC5953453

[CIT0005] Brand JM , Bracke JW, Britton LN, Markovetz AJ, Barras SJ. Bark beetle pheromones: production of verbenone by a mycangial fungus of *Dendroctonus frontalis*. J Chem Ecol. 1976:2(2):195–199. 10.1007/bf00987742

[CIT0006] Brown MS , Addesso KM, Baysal-Gurel F, Youssef NN, Oliver JB. Permethrin residual activity against ambrosia beetle (Coleoptera: Curculionidae: Scolytinae) attacks following field aging and simulated rainfall weathering. J Econ Entomol. 2020:113(5):2418–2426. 10.1093/jee/toaa186 32865196 PMC7564401

[CIT0007] Cavaletto G , Faccoli M, Ranger CM, Rassati D. Ambrosia beetle response to ethanol concentration and host tree species. J Appl Entomol. 2021:145(8):800–809. 10.1111/jen.12895

[CIT0008] Cavaletto G , Ranger CM, Reding ME, Montecchio L, Rassati D. Species-specific effects of ethanol concentration on host colonization by four common species of ambrosia beetles. J Pest Sci. 2023:96:833–843.

[CIT0009] Dobrin GC , Hammond RB. The antifeeding activity of selected pyrethroids towards the Mexican bean beetle (Coleoptera: Coccinellidae). J Kansas Entomol Soc. 1985:58:422–427.

[CIT0010] Dodds KJ , Miller DR. Test of nonhost angiosperm volatiles and verbenone to protect trap trees for *Sirex noctilio* (Hymenoptera: Siricidae) from attacks by bark beetles (Coleoptera: Scolytidae) in the northeastern United States. J Econ Entomol. 2010:103(6):2094–2099. 10.1603/EC10225 21309230

[CIT0011] Eisen L , Rose D, Prose R, Breuner NE, Dolan MC, Thompson K, Connally N. Bioassays to evaluate non-contact spatial repellency, contact irritancy, and acute toxicity of permethrin-treated clothing against nymphal *Ixodes scapularis* ticks. Ticks Tick-borne Dis. 2017:8:837–849. 28754599 10.1016/j.ttbdis.2017.06.010 PMC5665650

[CIT0012] Faulde M , Scharninghausen J, Tisch M. Preventive effect of permethrin-impregnated clothing to *Ixodes ricinus* ticks and associated *Borrelia burgdorferi* in Germany. Inter J Med Microbiol. 2008:298:321–324.

[CIT0013] Frank SD , Klingeman W III, White S, Fulcher A. Biology, injury, and management of maple tree pests in nurseries and urban landscapes. J Integr Pest Manage. 2013:4:B1–B14.

[CIT0014] Frank SD , Sadof CS. Reducing insecticide volume and nontarget effects of ambrosia beetle management in nurseries. J Econ Entomol. 2011:104(6):1960–1968. 10.1603/ec11124 22299358

[CIT0015] Fulcher A , Klingeman WE, Chong J-H, LeBude A, Armel GR, Chappell M, Frank S, Hale F, Neal J, White S. Stakeholder vision of future direction and strategies for southeastern US nursery pest research and extension programming. J Integr Pest Manage. 2012:3:D1–D8.

[CIT0016] Greco E , Wright MG. Ecology, biology, and management of *Xylosandrus compactus* (Coleoptera: Curculionidae: Scolytinae) with emphasis on coffee in Hawaii. J Integr Pest Manage. 2015:6:7.

[CIT0017] Gugliuzzo A , Biedermann PH, Carrillo D, Castrillo LA, Egonyu JP, Gallego D, Haddi K, Hulcr J, Jactel H, Kajimura H. Recent advances toward the sustainable management of invasive *Xylosandrus* ambrosia beetles. J Pest Sci. 2021:94:615–637.

[CIT0018] Hughes MA , Martini X, Kuhns E, Colee J, Mafra-Neto A, Stelinski L, Smith J. Evaluation of repellents for the redbay ambrosia beetle, *Xyleborus glabratus*, vector of the laurel wilt pathogen. J Appl Entomol. 2017:141:653–664.

[CIT0019] [IRAC] Insecticide Resistance Action Committee. The IRAC mode of action classification online; 2022 [accessed 22 Sep 2022]. (https://irac-online.org/mode-of-action/classification-online/).

[CIT0020] Joseph SV. Repellent effects of insecticides on *Stephanitis pyrioides* (Hemiptera: Tingidae) under laboratory conditions. Crop Prot. 2020:127:104985. 10.1016/j.cropro.2019.104985

[CIT0021] Joseph SV. Efficacy of cyantraniliprole trunk spray against ambrosia beetles on red maple bolts, 2021. Arthropod Manag Tests. 2022a:47(1):tsac008.

[CIT0022] Joseph SV. Effects of insect growth regulators on ambrosia beetles (Coleoptera: Curculionidae). J Entomol Sci. 2022b:57(3):380–393. 10.18474/jes21-73

[CIT0023] Klingeman WE , Bray AM, Oliver JB, Ranger CM, Palmquist DE. Trap style, bait, and height deployments in black walnut tree canopies help inform monitoring strategies for bark and ambrosia beetles (Coleoptera: Curculionidae: Scolytinae). Environ Entomol. 2017:46(5):1120–1129. 10.1093/ee/nvx133 28961948

[CIT0024] LeBude AV , White SA, Fulcher AF, Frank S, Klingeman WE III, Chong JH, Chappell MR, Windham A, Braman K, Hale F. Assessing the integrated pest management practices of southeastern US ornamental nursery operations. Pest Manage Sci. 2012:68:1278–1288. 10.1002/ps.3295 22517784

[CIT0025] Leufvén A , Bergström G, Falsen E. Interconversion of verbenols and verbenone by identified yeasts isolated from the spruce bark beetle *Ips typographus*. J Chem Ecol. 1984:10(9):1349–1361. 10.1007/BF00988116 24317586

[CIT0026] Lin H , Hoy CW, Head G. Olfactory response of larval diamondback moth (Lepidoptera: Plutellidae) to permethrin formulations. Environ Entomol. 1993:22(5):1096–1102. 10.1093/ee/22.5.1096

[CIT0027] Lindgren BS , Miller DR. Effect of verbenone on five species of bark beetles (Coleoptera: Scolytidae) in lodgepole pine forests. Environ Entomol. 2002:31:759–765.

[CIT0028] Lowery DT , Boiteau G. Effects of five insecticides on the probing, walking, and settling behavior of the green peach aphid and the buckthorn aphid (Homoptera: Aphididae) on potato. J Econ Entomol. 1988:81(1):208–214. 10.1093/jee/81.1.208

[CIT0029] Meyer DA , Shafer TJ. Permethrin, but not deltamethrin, increases spontaneous glutamate release from hippocampal neurons in culture. Neurotoxicology. 2006:27:594–603. 16678264 10.1016/j.neuro.2006.03.016

[CIT0030] Monterrosa A , Joseph SV, Blaauw B, Hudson W, Acebes-Doria AL. Ambrosia beetle occurrence and phenology of *Xylosandrus* spp. (Coleoptera: Curculionidae: Scolytinae) in ornamental nurseries, tree fruit, and pecan orchards in Georgia. Environ Entomol. 2022:51(5):998–1009. 10.1093/ee/nvac064 36000696

[CIT0031] Orsborne J , Banks SD, Hendy A, Gezan SA, Kaur H, Wilder-Smith A, Lindsay SW, Logan JG. Personal protection of permethrin-treated clothing against *Aedes aegypti*, the vector of dengue and Zika virus, in the laboratory. PLoS One. 2016:11:e0152805. 27187593 10.1371/journal.pone.0152805 PMC4871372

[CIT0032] Pitman G , Vité J. Aggregation behavior of *Dendroctonus ponderosae* (Coleoptera: Scolytidae) in response to chemical messenger. Can Entomol. 1969:101:143–149.

[CIT0033] Pitman G , Vité J, Kinzer G, Fentiman A Jr. Specificity of population-aggregating pheromones in *Dendroctonus*. J Insect Physiol. 1969:15(3):363–366. 10.1016/0022-1910(69)90282-0

[CIT0034] R Core Team. R: a language and environment for statistical computing. Vienna, Austria: R foundation for Stat. Comp.; 2022. (https://doi.org/https://www.R-project.org/).

[CIT0035] Rabaglia RJ , Dole SA, Cognato AI. Review of American *Xyleborina* (Coleoptera: Curculionidae: Scolytinae) occurring north of Mexico, with an illustrated key. Ann Entomol Soc Am. 2006:99:1034–1056.

[CIT0036] Ranger CM , Reding ME, Persad AB, Herms DA. Ability of stress‐related volatiles to attract and induce attacks by *Xylosandrus germanus* and other ambrosia beetles. Agr Forest Entomol. 2010:12:177–185.

[CIT0037] Ranger CM , Tobin PC, Reding ME, Bray AM, Oliver JB, Schultz PB, Frank SD, Persad AB. Interruption of the semiochemical-based attraction of ambrosia beetles to ethanol-baited traps and ethanol-injected trap trees by verbenone. Environ Entomol. 2013:42(3):539–547. 10.1603/EN13016 23726063

[CIT0038] Ranger CM , Schultz PB, Frank SD, Chong JH, Reding ME. Non-native ambrosia beetles as opportunistic exploiters of living but weakened trees. PLoS One. 2015:10(7):e0131496. 10.1371/journal.pone.0131496 26134522 PMC4489854

[CIT0039] Ranger CM , Schultz PB, Reding ME, Frank SD, Palmquist DE. Flood stress as a technique to assess preventive insecticide and fungicide treatments for protecting trees against ambrosia beetles. Insects. 2016a:7(3):40. 10.3390/insects7030040 27548230 PMC5039553

[CIT0040] Ranger CM , Reding ME, Schultz PB, Oliver JB, Frank SD, Addesso KM, Hong Chong J, Sampson B, Werle C, Gill S. Biology, ecology, and management of nonnative ambrosia beetles (Coleoptera: Curculionidae: Scolytinae) in ornamental plant nurseries. J Integr Pest Manage. 2016b:7:1–23.

[CIT0041] Ranger CM , Reding ME, Addesso K, Ginzel M, Rassati D. Semiochemical-mediated host selection by *Xylosandrus* spp. ambrosia beetles (Coleoptera: Curculionidae) attacking horticultural tree crops: a review of basic and applied science. Can Entomol. 2021:153:103–120.

[CIT0042] Reding M , Oliver J, Schultz P, Ranger C. Monitoring flight activity of ambrosia beetles in ornamental nurseries with ethanol-baited traps: influence of trap height on captures. J Environ Hortic. 2010:28(2):85–90. 10.24266/0738-2898-28.2.85

[CIT0043] Reding ME , Oliver JB, Schultz PB, Ranger CM, Youssef NN. Ethanol injection of ornamental trees facilitates testing insecticide efficacy against ambrosia beetles (Coleoptera: Curculionidae: Scolytinae). J Econ Entomol. 2013:106(1):289–298. 10.1603/ec12315 23448043

[CIT0044] Reding ME , Ranger CM. Attraction of invasive ambrosia beetles (Coleoptera: Curculionidae: Scolytinae) to ethanol-treated tree bolts. J Econ Entomol. 2020:113(1):321–329. 10.1093/jee/toz282 31693103

[CIT0045] Rieth JP , Levin MD. The repellent effect of two pyrethroid insecticides on the honey bee. Physiol Entomol. 1988:13(2):213–218. 10.1111/j.1365-3032.1988.tb00925.x

[CIT0046] Rivera MJ , Martini X, Conover D, Mafra-Neto A, Carrillo D, Stelinski LL. Evaluation of semiochemical based push-pull strategy for population suppression of ambrosia beetle vectors of laurel wilt disease in avocado. Sci Rep. 2020:10:1–12. 32060382 10.1038/s41598-020-59569-0 PMC7021720

[CIT0047] [UGA] University of Georgia. Georgia farm gate value report; 2020 [accessed 17 Sep 2022]. https://caed.uga.edu/content/dam/caes-subsite/caed/publications/annual-reports-farm-gate-value-reports/Farm%20Gate%20Report%202020.pdf.

[CIT0048] [USDA] United States Department of Agriculture. Census of Agriculture 2017; 2017 [accessed 17 Sep 2022]. (https://www.nass.usda.gov/Publications/AgCensus/2017/Full_Report/Volume_1,_Chapter_1_US/usv1.pdf).

[CIT0049] Weber B , McPherson J. Life history of the ambrosia beetle *Xylosandrus germanus* (Coleoptera: Scolytidae). Ann Entomol Soc Am. 1983:76:455–462.

[CIT0050] Yan Y , Yang Y, You J, Yang G, Xu Y, Huang N, Wang X, Ran D, Yuan X, Jin Y. Permethrin modulates cholinergic mini-synaptic currents by partially blocking the calcium channel. Toxicol Lett. 2011:201:258–263. 21251955 10.1016/j.toxlet.2011.01.009

