# Supplementary figures and images for: Effects of permethrin on ambrosia beetles (Coleoptera: Curculionidae: Scolytinae) in ornamental nurseries
Source: J Insect Sci. 2023 Jul 7;23(4):4. doi: 10.1093/jisesa/iead052 (PMC10327883; doi:10.1093/jisesa/iead052)

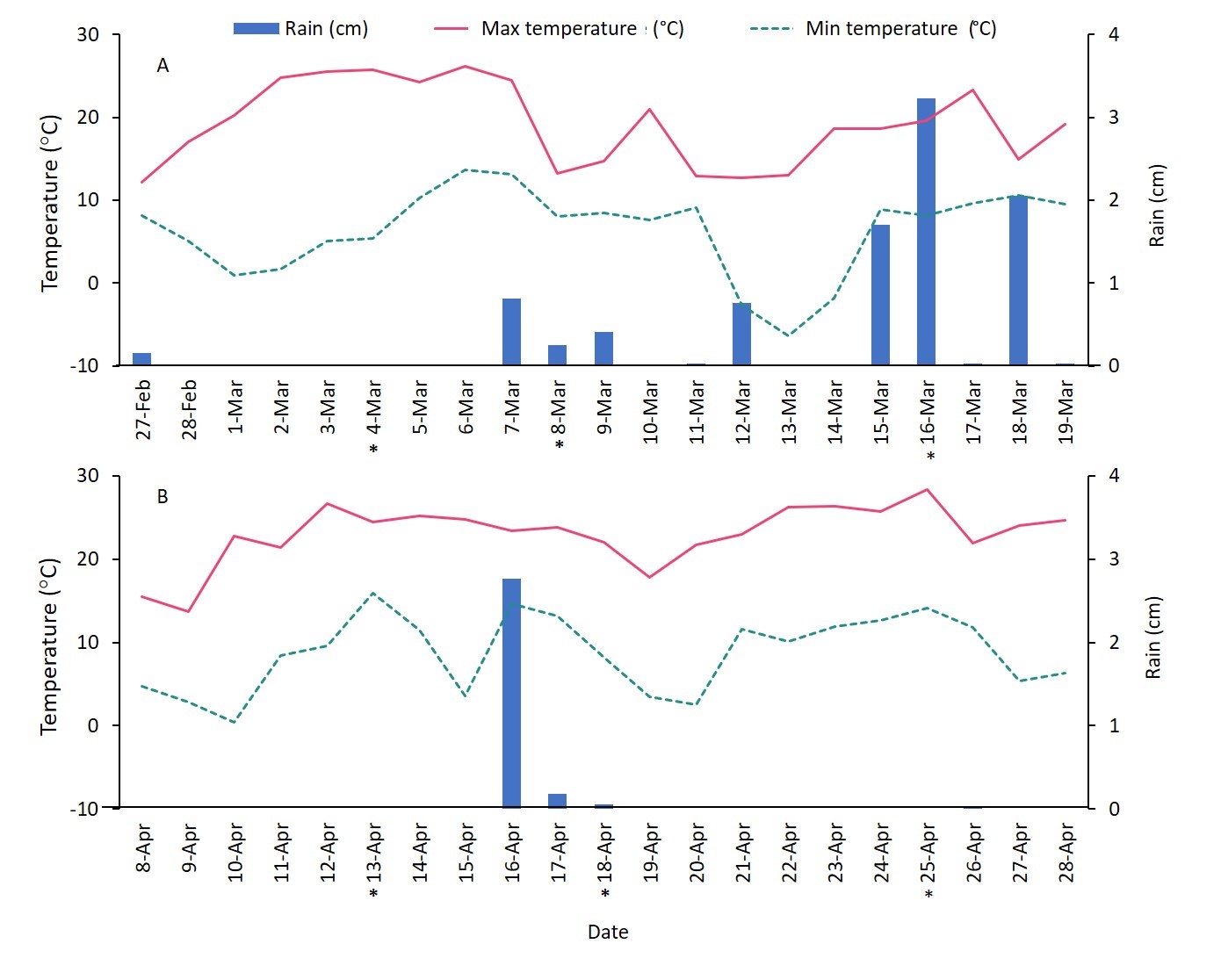

Supplement: iead052_suppl_Supplementary_Figure_S1 [file iead052_suppl_supplementary_figure_s1.jpeg]
